# Health Effects of Air Pollution in China

**DOI:** 10.3390/ijerph15071471

**Published:** 2018-07-12

**Authors:** Wenling Liu, Ziping Xu, Tianan Yang

**Affiliations:** 1 School of Management and Economics, Beijing Institute of Technology, Beijing 100081, China; liu_wenling@126.com; 2 Centre for Energy and Environmental Policy Research, Beijing Institute of Technology, Beijing 100081, China; 3 Sustainable Development Research Institute for Economy and Society of Beijing, Beijing 100081, China; 4 Beijing Key Lab of Energy Economics and Environmental Management, Beijing 100081, China; 5 Department of Statistics, University of Michigan, Ann Arbor, MI 48109, USA; pkuluke@gmail.com; 6 Yuanpei College, Peking University, Beijing 100871, China

**Keywords:** health effect, air pollution, hierarchical linear model

## Abstract

*Background* Rapid economic and social development in China has resulted in severe air pollution and consequent adverse impacts on society. The health effects of air pollution have been widely studied. *Methods* Using information from the China Health and Retirement Longitudinal Study (CHARLS) database, we established a hierarchical linear model combining pollution and socioeconomic and psychosocial variables to examine the effects of air pollution on public health in China. Local air pollution was characterized in multiple dimensions. *Results* The relationship of health to its determinants greatly differed between Eastern and Central/Western China. Higher education, higher income level, better life satisfaction, and long-term marriage were significantly associated with better health status among Chinese. In addition, regional healthcare resources were positively associated with the health of residents. As indicated by the hierarchical model with health as dependent variable, in Central/Western China, longest duration of good air quality in spring/summer was positively associated with health (estimated coefficient = 0.067, standard error = 0.026), while the mean Air Quality Index (AQI) in autumn/winter was inversely associated with health (estimated coefficient = −0.082, standard error = 0.031). Good air quality in the current study is defined as daily average AQI less than 35. *Conclusions* Duration (in days) of acceptable air quality was particularly important for improving public health. Future policies should target increased duration of good air quality while managing air pollution by controlling or decreasing severe air pollution.

## 1. Introduction

The adverse health effects of air pollution have generated considerable interest, and studies confirm that exposure to air pollution increases health risks, including adverse cardiovascular, respiratory, pulmonary, and other health-related outcomes [1,2,3]. The economic and social harms of air pollution arising from its negative effects on public health have been widely discussed, especially in China [4,5]. Unprecedented growth and development in China have had a substantial cost on the environment and pose a threat to public health. Indeed, air pollution is a major issue in China, and smog is increasingly frequent and severe in many cities. The Chinese government has developed several strategies to deal with regional air pollution, and public health is considered, in combination with environmental issues, as a national strategy. In 2016, the Central Committee of the Communist Party of China and State Council released the “Healthy China 2030 Plan”, which emphasizes improved management of health-related environmental problems.

Researchers have been widely interested in the health effects of environmental pollution. Numerous studies have examined the adverse health effects of air pollution using the exposure-response relationship [6,7,8,9]. These studies have mostly relied on mortality and morbidity data, due to easy accessibility and reliability of those health data. In addition, most of the previous studies focused on only a few health outcomes, such as cardiovascular and respiratory disease, for which the biological mechanism underlying the harmful effect of air pollution on the outcome is relatively clear. However, the effects on public health are not always reflected in disease mortality and morbidity data. The World Health Organization (WHO) defines good health as a state of complete physical, mental, and social well-being and not merely as the absence of disease or infirmity. In other words, health is a complex, comprehensive concept, and not all health impacts can be captured in morbidity or mortality data. Health status is a comprehensive judgement based on disease status and labor capacity. Numerous studies have analyzed the link between air pollution and public health, and the physical health of people has been a major focus in the literature [9,10,11]. Nevertheless, the impact of air pollution on other types of outcomes is unclear. For instance, air pollution can also damage mental health, and not many studies have considered the mental health impacts of air pollution.

Methods using the health production function or ER function cannot explain individual-level differences in health effects in the same exposure context. Concretely, a population group with a specific socioeconomic status might be more sensitive than other groups to air pollution. Econometric methods are widely used to examine the relationship between environmental pollution and public health and appear to be useful in both overcoming the bias seen in the above models and avoiding potential sources of inaccuracy (ER function). Although some research has examined both the physical and mental health impacts of environmental pollution, most studies of individual health rely on subjective individual self-assessment [12,13,14]. However, assessment of individual health status would benefit from a more rigorous scientific method. Population health is usually measured by examining the prevalence of chronic diseases [15,16,17], but this approach does not consider whether two persons had a similar health status when they received an identical diagnosis. Moreover, the air pollution status of regions is usually characterized, rather simply, by using the average concentration of pollutants (or air quality index). Unfortunately, the ‘average’ variable might obscure the specific characteristics of pollution, such as the distribution of days with severe pollution. 

To determine the effects of air pollution on public health, this study used a hierarchical linear model to examine information from the China Health and Retirement Longitudinal Study (CHARLS) database [18]. The analysis yielded precise assessments of individual health status by developing a comprehensive health metric for the Chinese participants. This metric was an inclusive assessment that included grip strength, lung function, balance, cognitive function, and impairments in physical and mental functions. Crude mean AQI is not an ideal indicator of the effects of air pollution on health; thus, we characterized local air pollution in multiple dimensions, and, in particular, deconstructed air pollution in relation to the duration and frequency of exposure. Analysis of this new perspective provides an important contribution to the literature. We begin with a review of the literature on the health effects of environmental pollution. Section 2 describes the data processing and other methods, Section 3 presents the results of the models, Section 4 contextualizes the results and identifies policy implications, and the final section offers our conclusion and discusses the study limitations. 

### Literature Review

The health effects of environmental pollution have been widely studied over time. In the 1980s, social scientists began to examine the environmental crisis and its related health effects, particularly in developing countries. Early research mostly considered the disease risks of chemical and water pollution and the potential effects on food safety [19]. The health effects of air pollution were not as obvious as the environmental degradation, but air pollution and its health effects are now a global concern [20]. 

Early studies of the impacts of environmental pollution on public health analyzed health as a kind of economic capital. The pioneering work of Grossman established the health production function model, which was later modified and improved by Cropper, and Gerking and Stanley [21,22,23]. The health production function views health as a durable capital stock that produces an output of healthy time, and views healthcare, lifestyle, environment, and heredity as the main factors affecting health status. Many subsequent studies conducted economic analyses of environmental pollution and public health by using this health production function approach [24,25,26].

The impact of air pollution on public health largely depends on the probability of exposure to pollution [27]. Massive studies examined the health harms of air pollution by using the exposure-response (ER) function, which calculates the number of health end-outcomes or change in death rate caused by a 1-unit increase in the concentration of a pollutant [5]. Accurate information on concentration–response relationships is essential when investigating such effects. Numerous studies [1,2,7,8,10,28,29,30] were undertaken to establish reliable ER functions in both developed and developing countries. These studies are the main body of evidence for the effects of air pollution on public health and generally confirm statistically significant relationships between levels of pollutants—such as particulate matter (PM) and ozone in ambient air—and mortality and other cardiopulmonary [6,7,8,31], cardiovascular, and respiratory health outcomes [9,10]. 

Evaluation of environmental health impacts has also been widely used to quantify the economic costs of environmental pollution [32,33,34,35], and some studies have focused on these issues in China [36,37,38,39]. Estimating these pollution costs is complicated due to the difficulty of assigning values for “non-market” impacts such as lost lives, biodiversity loss, and landscape degradation [5]. Identifying and applying the exposure-response relationship has enabled quantitative assessment of health impacts by focusing on the broader effects of environmental pollution. However, the reliability of the results has been questioned. Because the exposure-response relationship is used to determine estimates, its accuracy is paramount. Nevertheless, it treats audience objectives as homogeneous, even though indicators of environmental pollution, such as concentrations of pollutants, do not represent the exposure level of the target, and individual exposure time at a certain pollution level is unknown [12].

The econometric model is also widely used to estimate the health effects of environmental pollution. Econometric estimation examines causal relationships but does not need to be based on a mechanism of influence, such as the exposure-response relationship, and thus, can avoid the potential inaccuracies of the ER function. For example, recent studies have used the Huai River Policy in China to examine the health consequences of sustained exposure to air pollution. In an econometric model examining concentrations of total suspended particulates (TSP) in 90 cities from 1981 to 2000, Chen et al. found that space-heating policy in China had dramatic impacts on pollution and human health. Particulate concentrations north of the Huai River were 55% higher, which suggested a loss of more than 2.5 billion life-years among 500 million residents of Northern China during the 1990s [11]. A later study by Ebenstein examined the impacts of PM_10_ (coarse dust particles 2.5 to 10 micrometers in diameter) exposure from 1981 to 2012 and estimated that the Huai River Policy increased PM_10_ exposure to 41.7 μg/m^3^ and decreased life expectancy by 3.1 years in the area just north of the river [40]. Zhang used nationwide longitudinal survey data to investigate the effects of local air quality in China on public mental status and subjective well-being [41]. 

Methods using the health production function or ER function cannot explain individual-level differences in health effects in the same exposure context. Concretely, a population group with a specific socioeconomic status might be more sensitive than other groups to air pollution [42,43]. In addition, residents of an area with greater pollution do not necessarily have a higher exposure risk, because they might, for example, adopt preventive countermeasures [44]. Because of the disparities in socioeconomic status, the probability of exposure to pollution varies by group and individual. An increasing number of studies are examining disparities in health effects within groups [45]. Because environmental pollution harms the health of certain groups and may widen income inequality, which itself adversely affects health, such groups are vulnerable to the “Pollution-Health-Poverty Trap” [12].

To avoid the risk of inaccuracy associated with the ER relationship, we propose to use an econometric model. Most region-level studies examined the effects of contextual pollution on regional mortality and morbidity rates [11]. Zhang et al. and Cieza et al. developed individual-level estimates but principally focused on general health with an evaluation based on individually reported health scores [41,46]. In the present study, we estimated the health effects of air pollution by using econometric models that evaluated individual-level health metrics. 

## 2. Materials and Methods

### 2.1. Data

We used data from the 2015 wave of the Chinese Health and Retirement Longitudinal Study (CHARLS) [18]. The CHARLS are biannual, longitudinal, nationally representative surveys of adults aged 40 years or more. Both datasets are available for researchers after registration and can be obtained on the corresponding website. 

### 2.2. Variables

The WHO defines health as a state of complete physical, mental, and social well-being and not merely the absence of disease or infirmity [47]. In developing a comprehensive health metric for the Chinese participants, we considered an inclusive assessment, including the grip strength, lung function, balance, cognitive function, impairments in physical and mental functions [48], and difficulties in activities of daily living of each respondent. Using information collected from 34 self-reported items on the Health Status Questionnaire, we then calculated health scores by using the Polytomous Rasch Model [49,50,51] and analytic strategies of Banks et al. and Cieza et al. [46,52]. We tested for differential item functioning (DIF) for the survey and sex and age groups (age ≤64 vs. >64 yeas) by using iterative hybrid ordinal logistic regression, with change in the McFadden pseudo R^2^ (>0.02) as the DIF criterion [53,54]. The final health scores ranged from 0 (worst health) to 100 (best health). 

We collected air quality index (AQI) observations from 1643 sites monitoring air quality fine PM (PM_2.5_), SO_2_, NO_2_, and other pollutants around China in 2015, as indicated on real-time air quality websites [55]. Data from these websites are of high quality because they are downloaded directly from the Chinese Environmental Protection Agency. We calculated the daily means from hourly observations at each site and computed the city daily average from all sites in a city. Air pollution is heavier in winter and autumn because the height of the boundary layer is lower, thus making air pollution denser, and because of heating in northern China [56,57,58]. Crude mean AQI is not an ideal indicator of the effects of air pollution on health because people tend to go outside less on days with heavy pollution. The effects of air pollution on health are unclear for frequent short-term exposure to heavy pollution as compared with long-term exposure to light pollution. Therefore, we evaluated the health effects of air pollution in relation to the duration and frequency of exposure by using the following variables: (1) longest duration (in days) of good air quality in autumn/winter (SerGoodFW), (2) longest duration (in days) of poor air quality in autumn/winter (SerBadFW), (3) longest duration (in days) of good air quality in spring/summer (SerGoodSS), (4) longest duration (in days) of poor air quality in spring/summer (SerBadSS), (5) mean PM_2.5_ in autumn/winter (pmAvg_FW), and (6) mean PM_2.5_ in spring/summer (pmAvg_SS).

Demographic information related to health (household income, Gini coefficient, gender, education level, marital status, life satisfaction, and city and province of residence) was also analyzed. To optimize the fit of our final empirical model, we combined 10 levels of education into four categories (no formal education [illiterate], less than 6 years (elementary school), 6–9 years (middle school), 9–12 years (high school) and longer than 12 years (higher education), marriage status into four categories (married, married but not living with spouse, not married, widowed), and life satisfaction into five categories (completely satisfied = 5, very satisfied = 4, somewhat satisfied = 3, not very satisfied = 2, not at all satisfied = 1). Gender was classified as male and female.

The dataset was based on individual-level and city-level information. Health scores, gender, education, marital status, life satisfaction, and income were based on the individual level, while the others were based on the city level, including Gini coefficient and air pollution variables. There were 53 cities in the Western/central region and 35 cities in the Eastern region.

### 2.3. Development of Core Model

Our analysis focused on two levels of variables: health and socioeconomic factors at the individual level, and environmental pollution and socioeconomic factors at the regional level. The basic assumptions of the traditional linear model are linearity, normality, homogeneity of variance, and independence. The latter two assumptions may not apply to the hierarchical data structure used in this paper and explaining the data merely at the individual level would misrepresent the results. The advantage of using a hierarchical linear structure is that it can decompose overall health effects into a micro level (individual) and macro level (social environment). Therefore, a hierarchical linear model was used to differentiate the health effects of air pollution on individuals and cities [59]. The equation for the multilevel model is:

(1) $$y_{ij}=\beta_{0j}+\beta_{1j}AirQuality+\overset{k}{\underset{l=1}{\sum }}\beta_{k}Demographics_{kij}+\varepsilon_{ij}$$

where $y_{ij}$ is the health score for subject *i* in city j, and *k* is the demographic parameter. In the Equations (2) and (3):

(2) $$\beta_{0j}=\gamma_{00}+\mu_{0j}$$

(3) $$\beta_{1j}=\gamma_{10}+\mu_{1j}$$

$\gamma_{00}$ and $\gamma_{10}$ denote fixed effects for the intercept and air quality parameter, respectively. The model contains a random intercept $\mu_{0j}$ and random slope $\mu_{1j}$ for the air quality parameter, which means that the intercept and slope of our regression equation can vary by city. When expressed on one line, the equation is

health = education + gender + marital status + income.log + satisfaction + poll_var + Gini + Beds.per.log|city. (4)

## 3. Results

### 3.1. Demographic Characteristics and Health Status of Participants

Table 1 shows the characteristics of the participants (age, sex, education, and income) and health scores by region (Central/Western and Eastern China). The population of Central/Western China was healthier than that of Eastern China. The average health score of the participants was 50.3 (standard error [SE] = 10.1), 48.7% were male, and the average age was 64 years. Most were married (83.2%) and had finished elementary school (40.6%) or middle school (21.4%); only 4.6% had attended college or university. Average income was 2616.6 (SE = 10647.9) Chinese yuan. Most (57.4%) were unsatisfied with life. The longest duration of good and bad air quality in autumn and winter were 6.4 (SE = 5.7) and 10.4 (SE = 5.5) days, respectively. The average and 90th percentile of the Air Quality Index was 95.8 (SE = 27.8) and 165.7 (SE = 54.2). The number of hospital beds per 1000 persons was 5.30 (SE = 2.03). The Gini coefficient for all participants was 0.692 (SE = 0.075).

### 3.2. Empirical Results: Determinants of Individual Health Status

Health scores significantly differed in relation to sex, education level, marital status, life satisfaction, income, and the longest duration of good air quality in the area (Table 2). Health status was better for men, married respondents, and those with greater education, life satisfaction, higher salaries, and longer duration of good air quality in the area.

**Figure 1:**
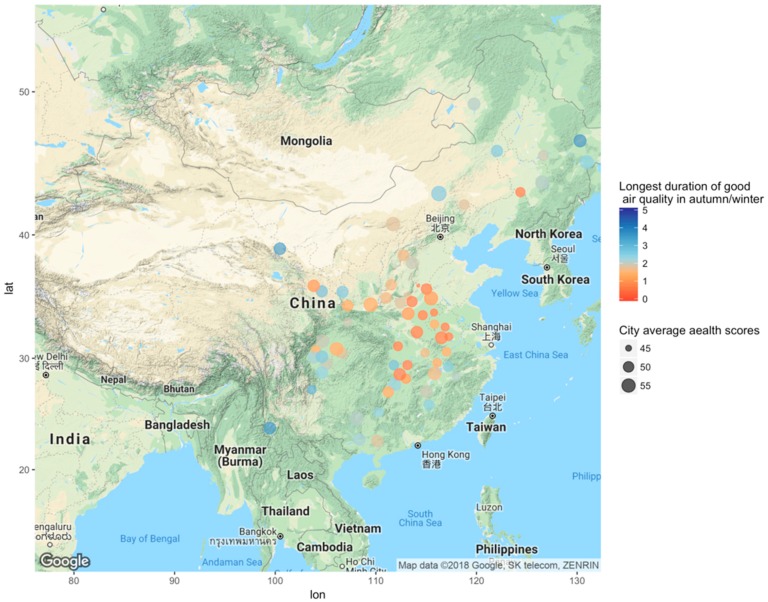
Correlation between health scores and longest duration of good air quality in autumn/winter in Central/Western China (The size of the dots represents population health in Central/Western China, and the color represents the longest duration of good air quality in autumn/winter in Central/Western China).

**Figure 2:**
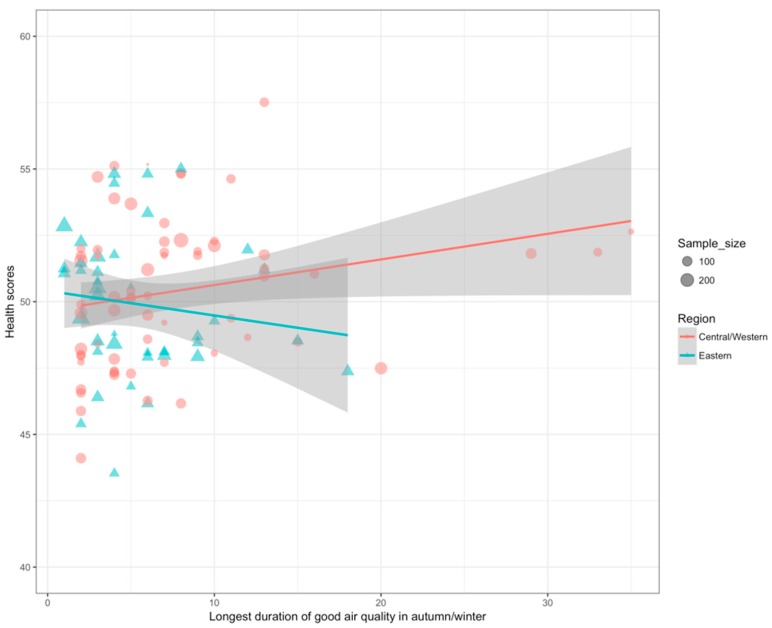
Correlation between health scores and longest duration of good air quality in autumn/winter in Central/Western China, stratified by region. (Solid lines indicate the fitted linear regression, and the grey regions indicate 95% confidence intervals. The dots represent cities in Central/Western China, and the triangles represent cities in Eastern China. The sizes of the dots and triangles represent sample size).

Correlation analysis using weighted sample sizes showed that the health impact of air pollution varied by region. In Central/Western China, longest duration of good air quality in autumn/winter was positively correlated with health scores; however, an inverse correlation was seen in Eastern China (Figure 1; Table 2 and Table 3). A married woman with a middle-school education, a salary around 992 CNY, and a satisfying life had a health score of 43–52. Health score increased with longer duration of good air quality in autumn/winter. Health scores were highest for participants in Xilinguolemeng and lowest for those in Shangrao. This situation was similar to Shanghai (Figure 2). 

### 3.3. Varied Health Effects of Air Pollution

Table 3 shows the results of univariate analysis of the effects of demographic characteristics and air quality on health scores, stratified by region. Table 4, Table 5 and Table 6 show the results of seven models simulating different variables representing air pollution. In most models, individual life satisfaction, income level, and regional medical resources (i.e., hospital beds per capita) were significantly positively associated with individual health. 

After adjusting for demographic factors (age, sex, education, marital status), life satisfaction, regional Gini coefficient, and the number of hospital beds, health score was significantly associated with two air pollution variables: longest duration of good air quality in spring/summer and mean PM_2.5_ in autumn/winter. Average AQI in autumn/winter had significant negative effects on public health in the Central/Western China (Table 4), and the longest duration of good air quality in spring/summer was significantly and positively associated with health in Central/Western China (Table 6). Participants (especially those in Central/Western China) who reported more education, being married, satisfaction with life, higher incomes, those who lived in cities with more hospital beds, and, in particular, those living in areas with better air quality in autumn/winter were healthier than other participants. The duration of good air quality days in spring/summer appears to be a useful indicator for any attempt to improve public health.

A sensitivity analysis of the results confirmed that they were robust. The details of the sensitivity analysis are presented in the Table S1 of supplementary materials.

## 4. Discussion

We used a hierarchical linear model and a database of individual health survey information to examine the effects of air pollution on public health. The simulation yielded precise calculations and evaluations of individual health status and was able to characterize local air pollution in multiple dimensions. 

The results of the health assessment showed regional disparities, but no obvious features were identified. Eastern areas had more medical resources and better climate conditions (coastal regions, in particular); however, the average health score for eastern areas was not higher than that of the central/western region. In Central/Western China, the longest duration of good air quality in autumn/winter was positively correlated with health scores. In general, population health in the central/western area was more sensitive to air quality. The relative lack of medical resources in these regions might increase this sensitivity. 

Our results confirmed that air pollution has significant adverse impacts on public health, especially in Central/Western China, which accords with many previous studies e.g., [1,2,3,11]. Past studies mostly examined the potential health effects of air pollution by analyzing average AOI or PM as an independent variable representing air pollution [11,40,41]. In the present study, high average AQI in autumn/winter was associated with significant negative impacts on public health. While crude mean AQI is not an ideal indicator of the effects of air pollution on health, we characterized local air pollution by deconstructing the duration and frequency of exposure. The results confirmed that good air quality is beneficial for health, and duration of fairly good air quality appears to be particularly important for improving public health. The previous Chinese policy target for managing air pollution focused mostly on controlling or decreasing the duration of heavy pollution; however, the present findings indicate that duration of fairly good air quality should also be considered in policy targets. 

This research provides a method for comprehensive evaluation of health, as it reflects both aspects of health, as represented by disease status and elements of mental health. This integrated health evaluation is a more accurate assessment of health status and better reflects the health effects of air pollution. Not surprisingly, individual life satisfaction, income level, and regional medical resources (hospital beds per capita) were significantly positively associated with individual health in most of the present models. These results are consistent with those of other health-related studies that included individual life satisfaction: subjective well-being, lifestyle-related attitudes, and life satisfaction as all important aspects in a comprehensive assessment of health status [60,61,62]. Zhang et al. reported adverse impacts of local air quality on public mental status and subjective well-being in Chinese [41], but few studies of air pollution and related human health outcomes have discussed this subjective aspect. As stated above, health is a complex concept influenced by numerous factors, including hereditary factors, emotional well-being, exercise, medical resources, and socioeconomic development. Health policy should be combined with environmental policy and urban development planning, and increased availability of medical and economic resources is especially important for improving health. In addition, policies that improve individual life satisfaction would benefit public health and help decrease the negative impacts of air pollution, especially in Chinese metropolises. 

This study has some limitations that warrant mention. Because of the lack of data, we elected to use a cross-sectional database for our analysis. Although the health data were collected from 2012, the nationwide environmental data covering PM_2.5_ emissions were accessed for the period since 2015. A future study should analyze the updated database and verify the present findings by using panel data. Because of limitations in the data, we did not analyze the cumulative or persistence of the health effects of air pollution. In addition, the time lag of the effects of air pollution on public health was difficult to integrate into the present models and has not yet been carefully studied. Such time lag is usually reflected by the incidences of diseases related to air pollution. However, this study comprehensively evaluated individual health and was thus, able to partially address this limitation.

## 5. Conclusions

Research has well documented health effects of air pollution. However, our study has built on a comprehensive measure of health in a national survey showing that the extent duration (in days) of acceptable air quality was particularly important for improving public health. Future policies should target increased duration of good air quality, medical resources and individual life satisfaction while managing air pollution by controlling or decreasing severe air pollution.

## Figures and Tables

**Table 1:** Demographic characteristics of participants by region.

	Total (*n* = 11953)		Central/Western China (*n* = 7473)		Eastern China (*n* = 4480)		*p*-Value *
Age (years)	64.1 ± 9.7		64.1 ± 9.7		64.3 ± 9.8		1.45 × 10^−4^
<65	6546	(54.8%)	4107	(55.0%)	2429	(54.4%)	
≥65	5407	(45.2%)	3366	(45.0%)	2041	(45.6%)	
Education							0.187
No education	3055	(25.5%)	1817	(24%)	1238	(28%)	
Elementary school	4850	(40.6%)	3081	(41%)	1769	(40%)	
Middle school	2558	(21.4%)	1624	(22%)	934	(21%)	
High school	935	(7.8%)	587	(8%)	348	(8%)	
Higher education	555	(4.6%)	364	(5%)	191	(4%)	
Sex							0.901
Male	5818	(48.7%)	3647	(49%)	2171	(48%)	
Female	6135	(51.3%)	3826	(51%)	2309	(52%)	
Marital status							0.0346
Married	9940	(83.2%)	6225	(83%)	3715	(83%)	
Married but not living with spouse	851	(7.1%)	540	(7%)	311	(7%)	
Not Married	68	(0.5%)	38	(1%)	30	(1%)	
Widowed	1094	(9.2%)	670	(9%)	424	(9%)	
Income (CNY) ^#^	2616.6 ± 10647.9		2608.9 ± 11469.9		2629.5 ± 9114.4		7.30 × 10^−4^
<1000	5893	(49.3%)	3647	(49%)	1464	(33%)	
≥1000	6060	(50.7%)	3826	(51%)	3016	(67%)	
Life satisfaction ^1^	3.4 ± 0.76		3.4 ± 0.77		3.4 ± 0.75		0.717
≤3	6863	(57.4%)	4212	(56%)	2651	(59%)	
>3	5090	(42.6%)	3261	(44%)	1829	(41%)	
Longest duration of good air quality in autumn/winter, days ^2,4^	6.4 ± 5.5		7.1 ± 6.2		5.1 ± 3.9		0.290
≤6	5946	(49.7%)	3168	(56%)	2778	(62.0%)	
>6	6007	(50.3%)	4305	(44%)	1702	(38.0%)	
Longest duration of bad air quality in autumn/winter, days ^2,4^	10.4 ± 5.7		11.3 ± 6.1		8.8 ± 4.6		9.05 × 10^−3^
≤8	5527	(46.2%)	2748	(56%)	2779	(62.0%)	
>8	6426	(53.7%)	4725	(44%)	1701	(40.0%)	
Average Air Quality Index ^4^	95.8 ± 27.8		91.9 ± 23.8		102.2 ± 32.5		0.779
≤90	5439	(45.5%)	3379	(56%)	2060	(45.9%)	
>90	6514	(54.5%)	4094	(44%)	2420	(54.1%)	
90th percentile of Air Quality Index ^4^	165.7 ± 54.2		158.5 ± 46.9		177.7 ± 62.9		0.720
≤160	6088	(50.9%)	3719	(49.7%)	2369	(52.8%)	
>160	5865	(49.1%)	3754	(50.3%)	2111	(47.2%)	
Number of hospital beds per 1000 persons ^4^	5.30 ± 2.03		5.35 ± 2.11		5.24 ± 1.91		0.548
≤5	6140	(51.4%)	2188	(48.8%)	4160	(55.6%)	
>5	5794	(48.5%)	2291	(51.1%)	3312	(44.3%)	
Gini coefficient ^4^	0.692 ± 0.075		0.694 ± 0.078		0.687 ± 0.069		0.263
≤0.7	6932	(58.1%)	2650	(59.1%)	4220	(56.4%)	
>0.7	5002	(41.9%)	1829	(44.3%)	3252	(43.5%)	
Health scores ^3^	50.3 ± 10.1		50.4 ± 4.43 × 10^−15^		50.1 ± 9.9		4.43 × 10^−15^

^#^ CNY: RMB, Chinese Yuan; ^1^ The range of the life satisfaction score is 1–5, higher scores indicate better life quality; ^2^ Cut-off at median value; ^3^ The range of the health score is 0–100, higher scores indicate better health status; ^4^ City-level variables, the t test or chi-square test was also used for the city level; * two-sample unpaired t test for continuous variables or Pearson chi-square test for discrete variables.

**Table 2:** Univariate analysis of the associations of participant demographic characteristics and air quality with health score by region.

	Central/Western China (*n* = 7473)		Eastern China (*n* = 4480)	
	Health Score	*p*-Value	Health Score	*p*-Value
Age (years)		0.3685		0.1835
<65	50.5 ± 10.2		49.9 ± 10.0	
≥65	50.3 ± 10.3		50.3 ± 9.9	
Education		<0.0001		0.0128
No education	49.98 ± 10.4		49.3 ± 9.7	
Elementary school	49.98 ± 10.0		50.1 ± 9.9	
Middle	50.94 ± 10.4		50.5 ± 10.3	
High school	51.43 ± 10.8		50.7 ± 9.5	
Higher education	52.46 ± 9.8		51.4 ± 9.8	
Sex		<0.0001		<0.0001
Male	52.3 ± 10.0		52.0 ± 9.7	
Female	48.6 ± 10.2		48.2 ± 9.8	
Marital status		<0.0001		<0.0001
Married	51.1 ± 10.1		50.6 ± 9.8	
Married but not living with spouse	50.0 ± 9.7		49.9 ± 10.1	
Not Married	41.0 ± 10.0		46.3 ± 10.0	
Widowed	45.3 ± 10.1		45.5 ± 9.9	
Income (CNY) ^#^		<0.0001		<0.0001
≥1000	52.4 ± 9.6		52.2 ± 9.4	
<1000	48.3 ± 10.4		47.9 ± 10.0	
Life Satisfaction		<0.0001		<0.0001
≤3	47.7 ± 9.1		47.9 ± 9.0	
>3	54.0 ± 10.5		53.2 ± 10.3	
Longest duration of good air quality in autumn/winter, days		0.0206		0.4781
≤6	49.4 ± 2.8		50.2 ± 2.7	
>6	51.0 ± 2.5		49.6 ± 2.7	
Longest duration of bad air quality in autumn/winter, days		0.4329		0.9202
≤8	50.7 ± 3.1		49.9 ± 3.0	
>8	50.0 ± 2.5		49.8 ± 2.6	
Average Air Quality Index		0.2601		0.6582
≤90	50.8 ± 3.6		49.7 ± 3.1	
>90	50.0 ± 2.9		50.1 ± 2.2	
90th percentile of Air Quality Index		0.5982		0.4745
≤160	50.5 ± 2.7		49.6 ± 3.0	
>160	50.2 ± 2.8		50.2 ± 2.3	
Number of hospital beds per 1000 persons		0.7641		0.8124
≤5	46.4 ± 10.04		49.1 ± 10.0	
>5	49.3 ± 10.04		50.2 ± 10.7	
Gini coefficient		0.8435		0.8673
≤0.7	47.0 ± 10.3		49.2 ± 9.6	
>0.7	48.3 ± 9.9		48.8 ± 10.9	

^#^ CNY: Chinese Yuan.

**Table 3:** Weighted sample size correlation between longest duration of good air quality in autumn/winter and health scores for each region.

	Overall	Central/Western	Eastern
*p*-Value	0.0030	<0.0001	0.0009
Correlation	0.0271	0.0522	−0.0491

**Table 4:** Multilevel models of health score and average/90th percentile of Air Quality Index in autumn/winter.

	Average AQI in Winter/Autumn				90th Percentile of AQI in Winter/Autumn			
	Central/Western China (*n* = 7473)		Eastern China (*n* = 4480)		Central/Western China (*n* = 7473)		Eastern China (*n* = 4480)	
	Estimates	*p*-Value	Estimates	*p*-Value	Estimates	*p*-Value	Estimates	*p*-Value
Fixed effects								
Intercept	3.919	0.661	11.167	0.114	9.511	0.333	22.137	0.013
Education								
No education	Reference		Reference		Reference		Reference	
Elementary school	1.228	0.023	1.040	0.017	1.224	0.023	0.707	0.347
Middle	−1.568	0.099	0.260	0.696	−1.531	0.107	1.641	0.089
High school	2.156	0.038	1.042	0.239	2.169	0.036	−2.275	0.177
Higher education	−1.022	0.021	−0.707	0.059	−1.015	0.023	−0.183	0.792
Sex								
Male	Reference		Reference		Reference		Reference	
Female	−2.576	0.000	−2.619	0.000	−2.575	0.000	−2.822	0.000
Marital status								
Married	Reference		Reference		Reference		Reference	
Married but not living with spouse	1.491	0.001	1.431	0.000	1.489	0.001	1.316	0.088
Not Married	−4.202	0.129	2.632	0.212	4.194	0.130	1.465	0.657
Widowed	0.741	0.749	−1.639	0.359	0.784	0.736	−5.648	0.045
Income (CNY) ^§,#^	1.558	0.000	1.340	0.000	1.561	0.000	0.859	0.000
Life Satisfaction	3.872	0.000	4.115	0.000	3.882	0.000	4.384	0.000
Average AQI in winter/autumn	−0.082	0.026	0.017	0.241	-	-	-	-
90th percentile of AQI in winter/autumn	-		-	-	−0.009	0.522	0.018	0.175
Gini coefficient	5.833	0.491	−0.931	0.890	−2.005	0.820	−14.313	0.379
Log number of beds per person	5.951	0.022	3.620	0.021	4.696	0.026	3.699	0.205

Note: the results are after adjustment for demographic characteristics and are stratified by region (only significant variables are included in model). * *p*-value < 0.05; ^§^ log transformation; ^#^ CNY: Chinese Yuan; -: not available.

**Table 5:** Multilevel models of health score and longest duration of good air/bad air/worst air in autumn/winter.

	Longest Duration of Good Air				Longest Duration of Bad Air				Longest Duration of Worst Air			
	Central/Western China (*n* = 7473)		Eastern China (*n* = 4480)		Central/Western China (*n* = 7473)		Eastern China (*n* = 4480)		Central/Western China (*n* = 7473)		Eastern China (*n* = 4480)	
	Estimates	*p*-Value	Estimates	*p*-Value	Estimates	*p*-Value	Estimates	*p*-Value	Estimates	*p*-Value	Estimates	*p*-Value
Fixed effects												
Intercept	6.468	0.433	25.681	0.034	8.842	0.360	21.820	0.036	Not Converge!		21.415	0.003
Education												
No education	Reference		Reference		Reference		Reference		Reference		Reference	
Elementary school	1.250	0.020	0.759	0.313	1.228	0.023	0.774	0.303			0.710	0.345
Middle	−1.442	0.128	1.679	0.081	−1.530	0.107	1.693	0.079			1.637	0.090
High school	2.259	0.029	−2.159	0.200	2.167	0.036	−2.092	0.214			−2.220	0.188
Higher education	−1.038	0.019	−2.814	0.789	−1.013	0.023	−0.193	0.781			−0.191	0.782
Sex												
Male	Reference		Reference		Reference		Reference		Reference		Reference	
Female	−2.575	0.000	−2.814	0.000	−2.575	0.000	−2.813	0.000			−2.821	0.000
Marital status												
Married	Reference		Reference		Reference		Reference		Reference		Reference	
Married but not living with spouse	1.503	0.001	1.295	0.095	1.488	0.001	1.277	0.099			1.299	0.093
Not Married	4.173	0.132	1.432	0.664	4.192	0.130	1.399	0.672			1.423	0.666
Widowed	0.666	0.775	−5.674	0.044	0.807	0.729	−5.675	0.044			−5.643	0.046
Income (CNY) ^§,#^	1.563	0.000	0.862	0.000	1.558	0.000	0.862	0.000			0.861	0.000
Life Satisfaction	3.868	0.000	4.376	0.000	3.884	0.000	4.377	0.000			4.384	0.000
Longest duration of good air in winter/autumn	0.105	0.246	−0.194	0.499	-	-	-	-				
Longest duration of bad air in winter/autumn					−0.052	0.467	0.099	0.640				
Longest duration of worst air in winter/autumn											0.674	0.120
Gini coefficient	−4.057	0.516	−8.102	0.669	−0.375	0.965	−3.089	0.849			−9.272	0.384
Log number of hospital beds per person	5.249	0.010	2.908	0.357	4.401	0.018	2.343	0.410			3.236	0.112

Note: the results are after adjustment for demographic characteristics and are stratified by region (only significant variables are included in model). * *p*-value < 0.05; ^§^ log transformation; ^#^ CNY: Chinese Yuan; -: not available.

**Table 6:** Multilevel models of health score and longest duration of good air/worst air in summer/spring.

	Longest Duration of Good Air in Spring/Summer				Longest Duration of Good Air in Spring/Summer			
	Central/Western China (*n* = 7473)		Eastern China (*n* = 4480)		Central/Western China (*n* = 7473)		Eastern China (*n* = 4480)	
	Estimates	*p*-Value	Estimates	*p*-Value	Estimates	*p*-Value	Estimates	*p*-Value
Fixed effects					Not Converge!			
Intercept	13.960	0.059	36.613	0.445			−28.276	0.361
Education								
No education	Reference		Reference				Reference	
Elementary school	0.063	0.889	0.910	0.261			0.886	0.302
Middle	−0.865	0.271	1.778	0.082			1.749	0.088
High school	1.417	0.119	−1.321	0.460			−1.450	0.382
Higher education	−0.970	0.016	−0.182	0.825			−0.162	0.848
Sex								
Male	Reference		Reference				Reference	
Female	−2.819	0.000	−2.992	0.000			−3.0	0.000
Marital status								
Married	Reference		Reference				Reference	
Married but not living with spouse	1.526	0.000	1.194	0.190			1.222	0.172
Not Married	3.586	0.197	0.277	0.939			0.292	0.911
Widowed	0.481	0.837	−6.711	0.028			−6.685	0.029
Income (CNY) ^§,#^	1.391	0.000	1.056	0.000			1.063	0.000
Life Satisfaction	3.684	0.000	4.238	0.000			4.256	0.000
Longest duration of good air in summer and spring	0.067	0.026	−0.632	0.144			-	-
Longest duration of worst air in summer and spring	-	-	-	-			0.928	0.105
Gini coefficient	−7.034	0.294	−30.684	0.231			−3.755	0.125
Log number of beds per person	4.424	0.012	4.908	0.706			14.951	0.168

Note: the results are after adjustment for demographic characteristics and stratified by region (only significant variables included in model); * *p*-value < 0.05; ^§^ log transformation; ^#^ CNY: Chinese Yuan; -: not available.

## Supplementary Materials

The following are available online at http://www.mdpi.com/1660-4601/15/7/1471/s1, Table S1: Multilevel models of health score and average/90th percentile of AQI in autumn/winter.
