# Supplementary material for: Health Effects of Air Pollution in China
Source: Int J Environ Res Public Health. 2018 Jul 12;15(7):1471. doi: 10.3390/ijerph15071471 (PMC6068713; doi:10.3390/ijerph15071471)
Supplement: Supplementary file 1 [file ijerph-15-01471-s001.pdf]

Supplementary Information to Health Effects of Air Pollution in China

Wenling Liu [1,2,3,4], Ziping Xu [5,6] and Tianan Yang [1,3,*]

[1] School of Management and Economics, Beijing Institute of Technology, Beijing 100081, China
[2] Centre for Energy and Environmental Policy Research, Beijing Institute of Technology, Beijing 100081, China
[3] Sustainable Development Research Institute for Economy and Society of Beijing, Beijing 100081, China
[4] Beijing Key Lab of Energy Economics and Environmental Management, Beijing 100081, China
[5] Department of Statistics, University of Michigan, Ann Arbor, MI 48109, USA
[6] Yuanpei College, Peking University, Beijing 100871, China
* Correspondence: tianan.yang@bit.edu.cn

1. Sensitivity Test

In figure 2, we see that some cities are of a much higher longest duration of good air quality in autumn/winter. Thus, it's reasonable to do a sensitivity test to make sure the findings are robust.

Our method is to remove first and last 10% samples according to the longest duration of good air quality in autumns/winter to see whether the coefficients are sensitive to the presence of outliers.

The following is the results of sensitivity test, by removing samples according to the longest duration of good air quality in autumns/winter and to AQI average in autumns/winter.

Table S1. Multilevel models of health score and average/90th percentile of AQI in autumn/winter.

	**Average AQI in Winter/Autumn**				**Longest Duration of Good Air**			
	**Central/Western China** (n = 7473)		**Eastern China** (n = 4480)		**Central/Western China** (n = 7473)		**Eastern China** (n = 4480)	
	**Estimates**	***p*-Value**	**Estimates**	***p*-Value**	**Estimates**	***p*-Value**	**Estimates**	***p*-Value**
**Fixed effects**								
Intercept	17.045	0.049	53.476	0.000	26.811	0.000	58.075	0.000
Education								
No education	Reference		Reference		Reference		Reference	
Elementary school	0.678	0.229	0.310	0.626	0.595	0.260	0.470	0.443
Middle	−0.913	0.259	−0.085	0.927	−0.826	0.279	−0.301	0.735
High school	0.575	0.551	−1.199	0.290	0.425	0.643	−1.047	0.347
Higher education	−0.140	0.798	0.051	0.932	0.247	0.635	0.393	0.503
Sex								
Male	Reference		Reference		Reference		Reference	
Female	−1.463	0.000	−0.707	0.135	−1.251	0.001	−0.908	0.004
Marital status								
Married	Reference		Reference		Reference		Reference	
Married but not living with spouse	1.639	0.004	4.439	0.000	1.528	0.005	3.967	0.000
Not Married	3.445	0.387	−4.810	0.296	3.147	0.412	−6.145	0.145
Widowed	3.866	0.225	−4.574	0.119	2.450	0.421	−4.388	0.114
Income (CNY) [§,#]	1.155	0.000	0.289	0.094	1.078	0.000	0.332	0.048
Life Satisfaction	4.440	0.000	4.596	0.000	4.322	0.000	4.755	0.000
**Average AQI in winter/autumn**	−0.085	0.033	-0.01	0.355	-	-	-	-
**90th percentile of AQI in winter/autumn**	-		-	-	0.041	0.803	-0.094	0.630
Gini coefficient	7.149	0.252	3.06	0.749	4.241	0.537	−5.403	0.590
Log number of beds per person	2.612	0.178	−1.93	0.421	2.935	0.041	−2.135	0.397

Note: the results are after adjustment for demographic characteristics and are stratified by region (only significant variables are included in model); *p-value < 0.05; § log transformation; # CNY: Chinese Yuan; -: not available.

Table S1 is the coefficients of four hierarchical linear model with samples removing first and last

10% according to AQI average and longest duration of good air quality in autumn and winter. Directions and significance of coefficients remain unchanged, which passes the sensitivity test.
